# Exploring the genetic prediction of academic underachievement and overachievement

**DOI:** 10.1038/s41539-024-00251-9

**Published:** 2024-06-01

**Authors:** Kaito Kawakami, Francesca Procopio, Kaili Rimfeld, Margherita Malanchini, Sophie von Stumm, Kathryn Asbury, Robert Plomin

**Affiliations:** 1 Social, Genetic and Developmental Psychiatry Centre, Institute of Psychiatry, Psychology and Neuroscience, King’s College London, London, UK; 2 Department of Psychology, Royal Holloway, University of London, London, UK; 3 School of Biological and Behavioural Sciences, Queen Mary University of London, London, UK; 4 Department of Education, University of York, York, UK

**Keywords:** Human behaviour, Human behaviour

## Abstract

Academic underachievement refers to school performance which falls below expectations. Focusing on the pivotal first stage of education, we explored a quantitative measure of underachievement using genomically predicted achievement delta (GPAΔ), which reflects the difference between observed and expected achievement predicted by genome-wide polygenic scores. We analyzed the relationship between GPAΔ at age 7 and achievement trajectories from ages 7 to 16, using longitudinal data from 4175 participants in the Twins Early Development Study to assess empirically the extent to which students regress to their genomically predicted levels by age 16. We found that the achievement of underachievers and overachievers who deviated from their genomic predictions at age 7 regressed on average by one-third towards their genomically predicted levels. We also found that GPAΔ at age 7 was as predictive of achievement trajectories as a traditional ability-based index of underachievement. Targeting GPAΔ underachievers might prove cost-effective because such interventions seem more likely to succeed by going with the genetic flow rather than swimming upstream, helping GPAΔ underachievers reach their genetic potential as predicted by their GPS. However, this is a hypothesis that needs to be tested in intervention research investigating whether GPAΔ underachievers respond better to the intervention than other underachievers. We discuss the practicality of genomic indices in assessing underachievement.

## Introduction

Underachievement in school is costly to society and to the children who fail to maximize their potential to flourish^1–3^. Academic underachievement refers to school performance that falls below expectations, typically assessed using previous achievement or tested ability^4,5^. Identifying underachieving children enables personalized efforts to help improve their performance. However, the process of ascertaining a student’s ability, and by extension, their predicted achievement, can be difficult. While previous achievement is the best predictor of future achievement, reliance on past observations delays the identification of underachieving students until they have fallen significantly behind. Intelligence tests have also been used to predict academic achievement—in fact, that is what they were originally designed for—but their prediction early in life, when the scope for effective interventions is the greatest, is limited (Davey^6^, p. 657). For example, intelligence assessed at age 2 or 3 predicts just 4% of the variance in intelligence at age 16, making it of limited value for prediction early in life, as compared to age 9, which predicts 20% of the variance at age 16^7^.

The DNA revolution has made it possible to predict individual differences in educational achievement from DNA rather than from measures of ability or previous achievement^8^. Genome-wide association (GWA) studies testing the association between hundreds of thousands of DNA differences across the genome have identified thousands of single-base pair differences in inherited DNA sequence that are associated with common disorders and complex traits^9^. Although the largest effect sizes are extremely small, often less than 0.1%, the effects of genetic variants associated with a trait as assessed in GWA studies can be aggregated in genome-wide polygenic scores (GPS)^10^.

A unique feature of GPS is their ability to serve as an early warning system because inherited DNA differences do not change systematically after conception and are stable throughout the lifespan. In other words, GPS can be used to predict school achievement just as well from infancy as from the school years. By comparison, in terms of predicting achievement at the end of secondary school, achievement tests can only predict from the school years; g can predict in early childhood, but its prediction is weak. In contrast, the prediction from GPS is exactly the same whether DNA was obtained at birth or in adolescence. An additional benefit of GPS is that, unlike achievement and ability measures, GPS are not subject to training, practice, or anxiety effects. While polygenic scores cannot serve as diagnostic instruments on their own (because they are continuous indices of risk), their use has been proposed for ‘predictive enrichment’- that is identifying which populations are most likely to benefit from a given treatment^11^.

Currently, GPS can predict up to 16% of the variance in educational achievement—a four-fold increase compared to g in infancy^7,12^. In the present study, we create a polygenic score based on the latest GWA study of educational attainment (years of schooling), EA4^13^. The EA4 GPS is currently the most powerful GPS predictor of education-related traits because this GWA study included three million participants in its discovery sample (Okbay et al.^13^). Although EA4 relies on the crude index of years of schooling, educational attainment represents the culmination of the educational process and incorporates all the traits needed for educational success, not just *g* but also personality traits such as conscientiousness and motivation as well as mental health. There are other education-related GPSs—for example, for IQ (Savage et al.^14^), cognitive performance (Lee et al.^15^), self-reported math ability (Lee et al.^15^), and math attainment (Lee et al.^15^), which we use in a multi-polygenic score approach^16^, even though they have been shown to add little to the predictive power of EA4^17^.

The present paper explores the concept of genomically predicted achievement delta (GPAΔ), which reflects the difference between children’s observed academic achievement and their expected achievement as indexed by the EA4 GPS. Specifically, we explore the extent to which students’ discrepancies between the EA4 GPS and observed achievement at age 7 (GPAΔ) regress towards their genomically predicted levels of achievement at ages 9, 12, and 16. Construction of these difference scores are described in Methods. Although the statistical phenomenon of regression to the mean predicts these GPAΔ achievement trajectories during the school years, our goal was to track these changes empirically. Moreover, in follow-up analyses, we intend to investigate the antecedents and sequelae of individual differences in these GPAΔ trajectories. As a first step in this direction, in the present paper we present estimates of the heritability of GPAΔ.

From a sample of 4175 genotyped UK individuals, we calculate difference scores at age 7, and chart their educational trajectories as longitudinal slopes at ages 9, 12 and 16. We are interested in the extent to which children who are underachieving at age 7 experience increases in achievement, in line with their genomic predictions as indexed by the EA4 GPS, and the extent to which overachievers experience a decline in achievement. A unique feature of the EA4 GPS is that it was created to assess adult educational attainment so that GPAΔ based on achievement at age 7 represents the extent to which achievement at age 7 differs from the genomic prediction of adult educational attainment. In contrast, traditional indices of underachievement use intelligence, a concurrent measure of ability, to assess predicted achievement.

**Fig. 1:**
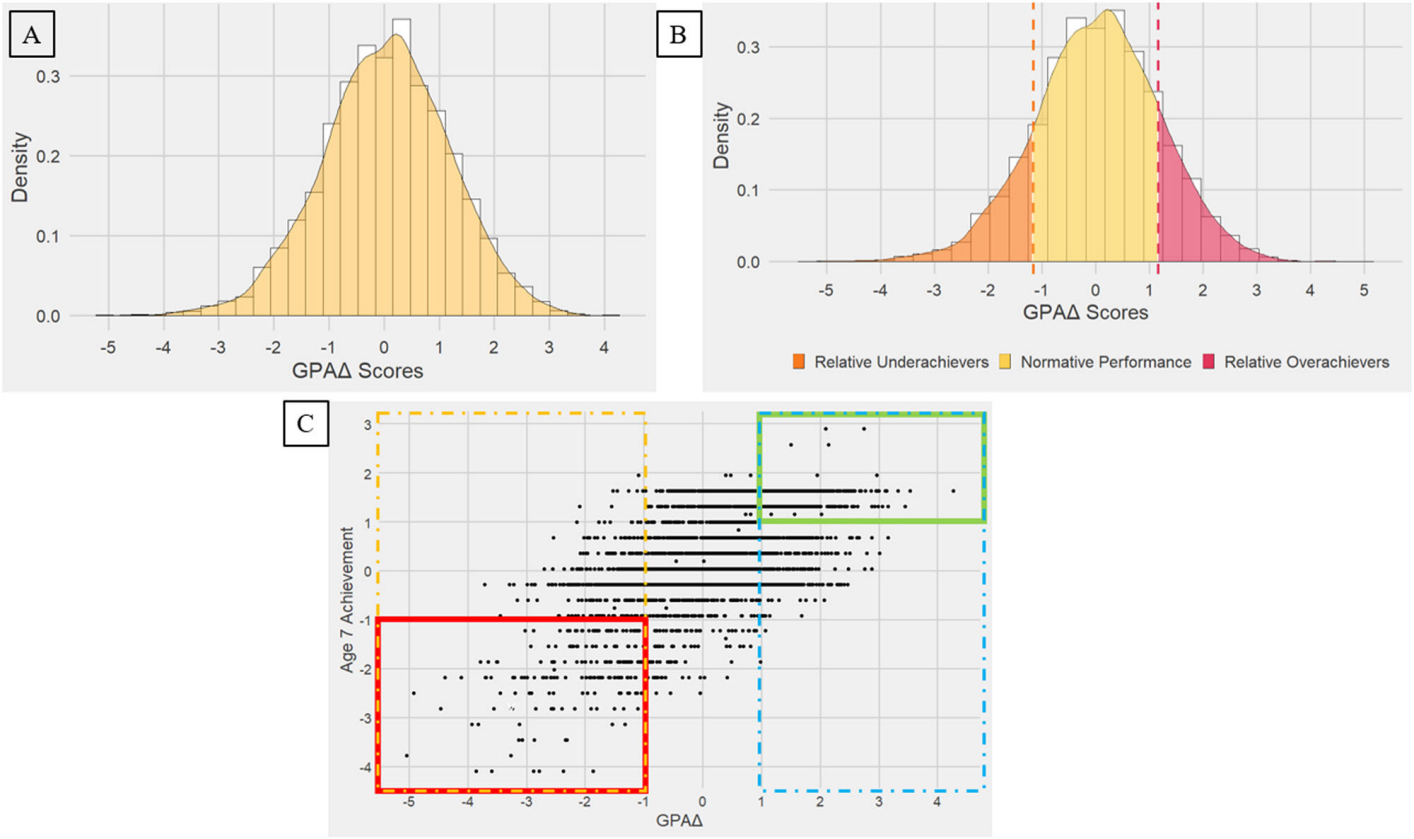
Sample selection of underachievement and overachievement groups. **A** depicts the distribution of GPAΔ scores (Achievement – GPS) for our entire sample. GPAΔ scores are normally distributed around a mean of zero. Negative GPAΔ scores indicate relative underachievement, while positive GPAΔ scores indicate relative overachievement **B**. We call these relative groups because their achievement scores will vary widely. That is, a student could be in the relative underachievers group despite having a relatively high achievement score. In **C**, we have plotted GPAΔ against age 7 achievement scores. The dashed yellow and blue lines denote relative underachievers and overachievers, respectively. We select a subset of underachievers with low achievement scores (red box) and a subset of overachievers with high achievement scores (green box), which we refer to as absolute underachievers and absolute overachievers, respectively.

After calculating GPAΔ, we analyzed our entire sample to investigate GPAΔ across the full range from underachievement to overachievement, as shown in Panel A of Fig. 1. We then focused on two subgroups from our total sample who have extreme GPAΔ scores. The first subsample, which we will call *relative* underachievers and overachievers, are those whose GPAΔ scores are 1 SD below and 1 SD above the GPAΔ mean, respectively (Panel B). We also considered a subset of relative underachievers and overachievers, which we call *absolute* underachievers and overachievers, whose achievement scores are 1 SD below and 1 SD above the achievement mean (Panel C).

While educational interventions for reversing underachievement have been moderately successful in improving school performance, a meta-analysis of 53 studies revealed substantial heterogeneity in effect sizes of treatment outcomes^18^. Interventions targeting gifted underachievers have particularly been unsuccessful^19^. Interestingly, one of the strongest moderators of intervention success is age: interventions delivered early in life are more likely to be successful^18^. Thus, screening for GPAΔ could eventually be a valuable early warning system, helping educators identify underachievers early in development.

Targeting GPAΔ might also prove cost-effective because such interventions seem more likely to succeed by going with the genetic flow rather than swimming upstream^8^, helping GPAΔ underachievers to reach their genetic potential as predicted by their GPS. However, this is a hypothesis that needs to be tested in intervention research investigating whether GPAΔ underachievers respond better to interventions than other underachievers. Moreover, until a GWA study is done on GPAΔ per se rather than on educational attainment or achievement, GPAΔ will require that early school achievement is assessed so that the difference between GPS (genomically expected achievement) and observed achievement can be obtained. Nonetheless, it is interesting to explore the educational trajectories through primary and secondary school of GPAΔ constructed on the basis of GPS at age 7, which is the rationale for the present study.

GPAΔ seems impractical now because it requires DNA, genotyping, and the creation of GPS. However, the rise in direct-to-consumer DNA testing suggests a future where GPAΔ becomes more accessible. At least 27 million people have paid direct-to-consumer DNA testing companies for this service^20^, and these companies are increasingly marketing their product to encourage parents to test their children^21^. National and private health systems are also beginning to use DNA testing to pivot from curing disorders to preventing them^8^. Once genotyping is available by whatever means, it will be possible to create GPS for educationally relevant traits, a process that is becoming routinized^22^.

Three other introductory issues should be mentioned. First, we acknowledge that many other factors could be involved in underachievement such as motivation, self-regulation, conscientiousness, and family environment^18,23^, which could be used together to maximize prediction. However, the goal of the present paper is to examine the extent to which underachievement can be usefully defined using genetics, in the same manner in which it is currently defined using intelligence. That is, while we acknowledge the importance of other factors, we are interested in exploring the use of GPS and early achievement to chart trajectories of underachievement during the school years. Nonetheless, we compare our results for GPAΔ to those for underachievement defined using g and assess the extent to which g and GPAΔ jointly predict achievement.

A second issue is development. Underachievement can emerge at different stages of development^24^. For some it may emerge in early school years, while for others it begins in middle school^25^. In the present study, we chose to focus on identifying underachievement and overachievement in the early school years because we are interested in how early differences in GPAΔ affect subsequent trajectories in educational achievement.

A third issue is domain-specific underachievement. Although the underachievement literature has broadly focused on failures to maximize potential in general achievement, there has been growing interest in understanding domain-specific underachievement, particularly within mathematics and reading^26,27^. In addition to creating general achievement indices of GPAΔ, we also constructed their domain-specific equivalents (i.e., English – GPS, Mathematics – GPS), in both cases assessing ability using the EA4 GPS. Finally, we compared our primary results for GPAΔ to those using a traditional index of underachievement (i.e., achievement – *g*), which we call cogΔ.

## Results

The primary goal of our analyses was to chart the course of GPAΔ at age 7 longitudinally in terms of achievement during the school years at ages 9, 12 and 16. Put simply, we explored the extent to which genomically predicted achievement discrepancies, calculated at age 7, reduced during the school years at 9, 12 and 16. We used participants from the Twins Early Development Study (TEDS), a nationally representative cohort of twins born between 1994 and 1996 in England and Wales (see Methods). Descriptive statistics of all measured variables are presented in Supplementary Table 1.

In overview, after calculating GPAΔ scores, we conducted analyses of GPAΔ and achievement slopes separately, followed by our main analyses relating GPAΔ to achievement slopes for the whole sample and for relative and absolute underachievers and overachievers. We then explored a multi-GPS approach, separate analyses of English and mathematics, and comparisons with cogΔ.

### Genomically predicted achievement discrepancies (GPAΔ)

We calculated Genomically-Predicted Achievement Discrepancy (GPAΔ) scores for each individual at age 7 by subtracting their standardized genome-wide polygenic score (GPS) from their standardized observed achievement at age 7 (see Methods for details on achievement measures). Polygenic scores are created based on individual SNP effect size estimates obtained from genome-wide association (GWA) studies. In the present study, we created a polygenic score based on the latest GWA meta-analysis of educational attainment (years of schooling), EA4 (Okbay et al.^13^). In our study, EA4 has a zero-order correlation of 0.28 with educational achievement at age 7, 0.26 at age 12 and 0.43 at age 16 (See Supplementary Table 12 for pairwise correlations between all measured variables). Subsequently, we added four other GPS: IQ (Savage et al.^14^), cognitive performance (Lee et al.^15^), self-reported math ability (Lee et al.^15^), and math attainment (Lee et al.^15^), in a multi-polygenic score approach^16^ in order to maximize genotypic prediction.

Although there are many quantitative genetic analyses of educational achievement, we are not aware of any genetic analyses of GPAΔ. For this reason, we conducted twin analyses of GPAΔ using the TEDS twins (see Methods). GPAΔ at age 7 correlates 0.57 with achievement at age 7, suggesting that they could show somewhat different aetiological influences. In addition, although GPAΔ subtracts the GPS for educational attainment from achievement at age 7, it does not necessarily follow that the heritability of GPAΔ will be low because the GPS correlates only 0.28 with achievement at age 7 (but 0.43 with achievement on national exams administered at age 16).

**Fig. 2:**
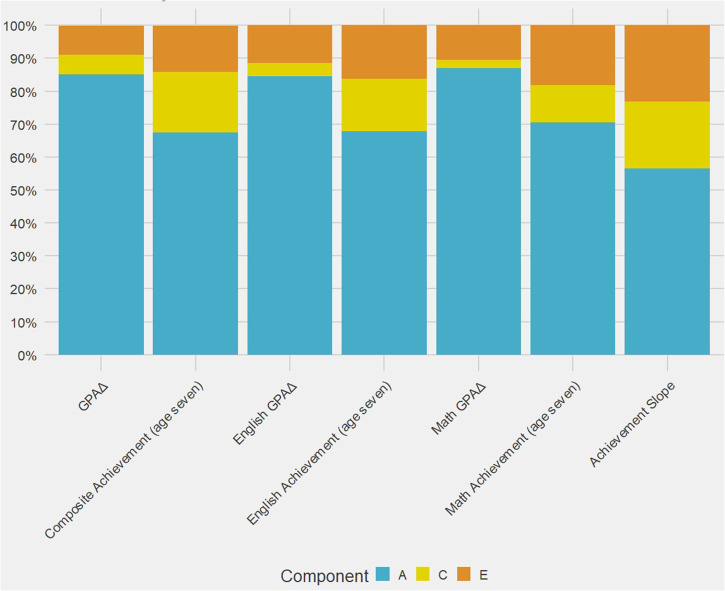
Twin ACE estimates for GPAΔ and related traits. Univariate twin model-fitting estimates of additive genetic variance **A**, common or shared environmental variance **C** and nonshared environmental variance **E** for GPAΔ and related traits.

Figure 2 displays twin estimates of additive genetic variance (A), common or shared environmental variance (C) and non-shared environmental variance (E). Heritability, the proportion of phenotypic variance explained by genetic variance, is strikingly high for GPAΔ (85%), higher than for general achievement itself at age 7 (67%). Heritabilities are similarly high for GPAΔ based on mathematics alone (87%) and English alone (85%), which are also higher than mathematics achievement (71%) and English achievement (68%). The last column in Fig. 2 indicates that the heritability of achievement slopes (57%) is slightly lower than the heritability of achievement (67%). Twin intraclass correlations and parameter estimates with confidence intervals are shown in Supplementary Table 2.

### Achievement slopes

We computed achievement composites by averaging standardized teacher-rated national curriculum scores in math, science, and English at the Key Stage ages of 7, 9, and 12. For age 16, we obtained GCSE scores, a standardized examination taken at the end of compulsory schooling in the UK, to construct our achievement composites (See Methods). To create *individual* scores representing changes in achievement over time, we calculated a line of best fit using age (7, 9, 12, and 16) as the x-variable and standardized achievement as the y-variable for each participant separately. The slope from the resulting line of best fit, which we call achievement slopes, was then extracted for each participant. We then mean standardized the achievement slope variable (mean = 0, SD = 1) in our analyses for ease of interpretation.

Achievement slopes for individuals whose achievement increases on average during the school years will be positive, average declines in achievement will be negative, and students whose achievement shows no average increase or decrease, perhaps by going up and down over the school years, will be near zero. Thus, when we refer to achievement slopes (calculated in relation to achievement z-scores for each participant), we are referring to relative achievement. Prior to running analyses we first used the median absolute deviation (MAD) method for outlier removal of achievement slope scores, described in Eq. (1),

1 $${MAD}={median}\left(\left|{x}_{i}-{median}\left(x\right)\right|\right)$$

such that ${x}_{i}$ is an individual observation.

### Associations between GPAΔ scores and achievement slopes

**Fig. 3:**
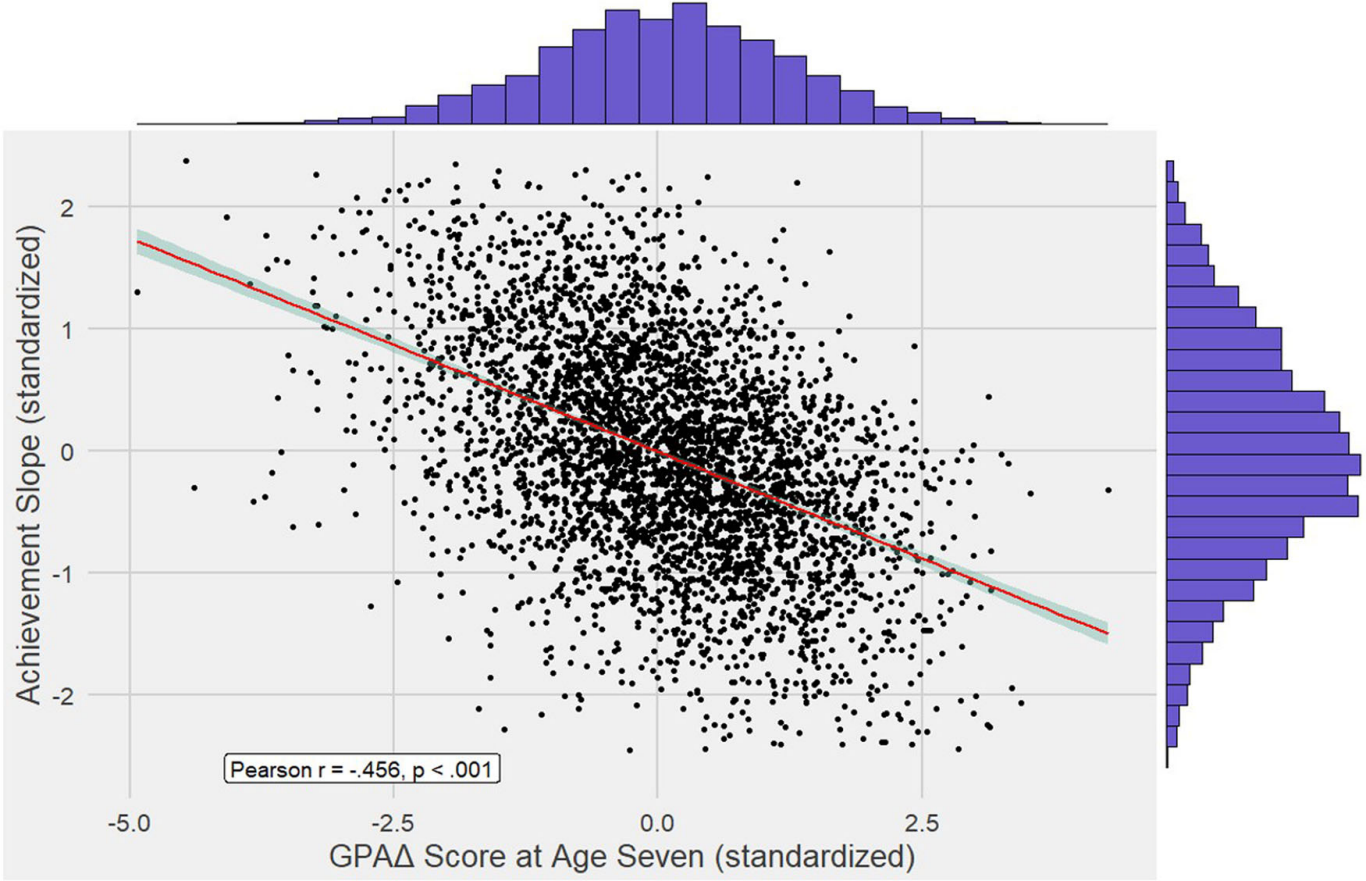
Scatterplot of GPAΔ scores and achievement slopes. Scatterplot for the correlation of −0.456 between GPAΔ scores (calculated as observed achievement – GPS) at age 7 and achievement slopes for each individual across ages 7, 9, 12 and 16 with marginal densities plotted in blue. Positive achievement slopes indicate increases in achievement over time, while negative slopes indicate decreases in achievement over time.

We correlated GPAΔ scores at age 7 with achievement slope scores, which index trajectories of change in achievement across ages 7, 9, 12 and 16. Figure 3 shows the normal distributions of GPAΔ and achievement slopes and a scatterplot of the correlation between them. The correlation was negative (*r*(4173) = −0.456, ${\text{CI}}_{95 \% }$[−0.480, −0.432], *p* < 0.001; Fig. 2) and explained 20.8% of the variance of achievement slopes. This negative correlation indicates that students whose achievement at age 7 was discrepant from their polygenic score moved towards their genomic trajectory at ages 9, 12, and 16. To calculate the extent to which participants regressed towards their genomically predicted levels, we first computed regression percentages using the formula in Eq. (2),

2 $$\left(1-\frac{{GPA}\Delta \,{\rm{at}}\; {\rm{age}}16}{{GPA}\Delta \,{\rm{at}}\; {\rm{age}}7}\right)* 100$$

and obtained the 5% trimmed mean to account for outliers. We found that participants, on average, regressed to 38.4% of their genomically predicted levels of achievement at age 16. In other words, GPAΔ underachievers progressed more and GPAΔ overachievers progressed less during their school years.

**Fig. 4:**
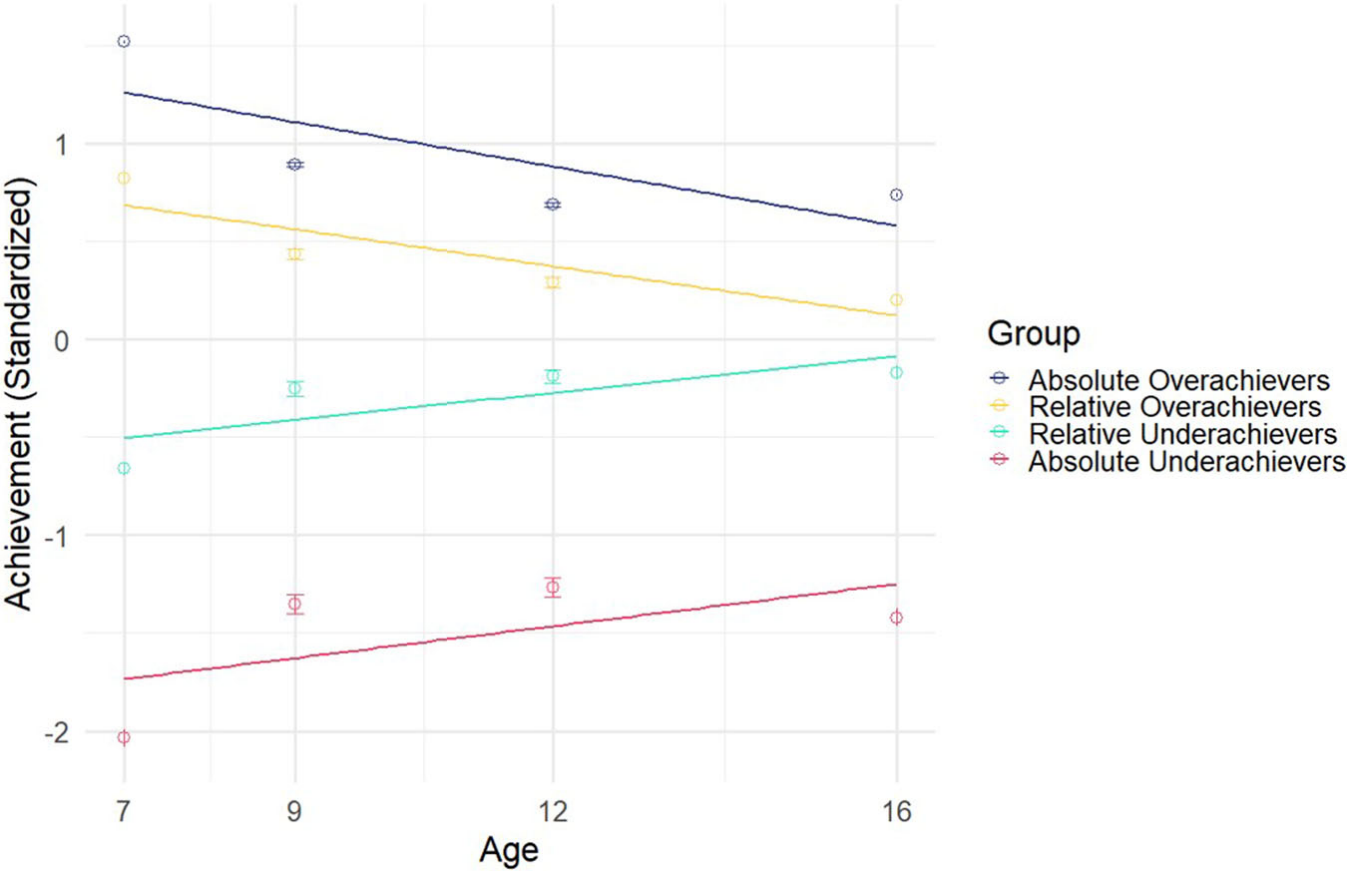
Scatterplot of average achievement over time for relative and absolute GPAΔ groups. Scatterplot of average achievement over time for relative and absolute GPAΔ groups (SE mean plotted in error bars). Group means of achievement are plotted across ages 7, 9, 12, and 16. Absolute overachievers regressed to 45.3% of their genomic prediction; relative overachievers regressed 38.9%; relative underachievers regressed 28.4%; absolute underachievers regressed 30.6%.

These results can be understood more readily by focusing on the extremes of GPAΔ underachievement and overachievement (Fig. 4). When examining relative and absolute GPAΔ groups, we found that all groups, on average, regressed substantially towards their genomically predicted levels of school achievement at age 16. By design, the absolute GPAΔ overachievers and underachievers had more extreme achievement scores than the comparable relative GPAΔ groups, but both absolute and relative groups returned to 28-45% of their predicted levels (Fig. 4). It is noteworthy that most of the change occurs by age 9, suggesting the importance of the early school years. See Supplementary Table 1 for descriptive statistics.

**Fig. 5:**
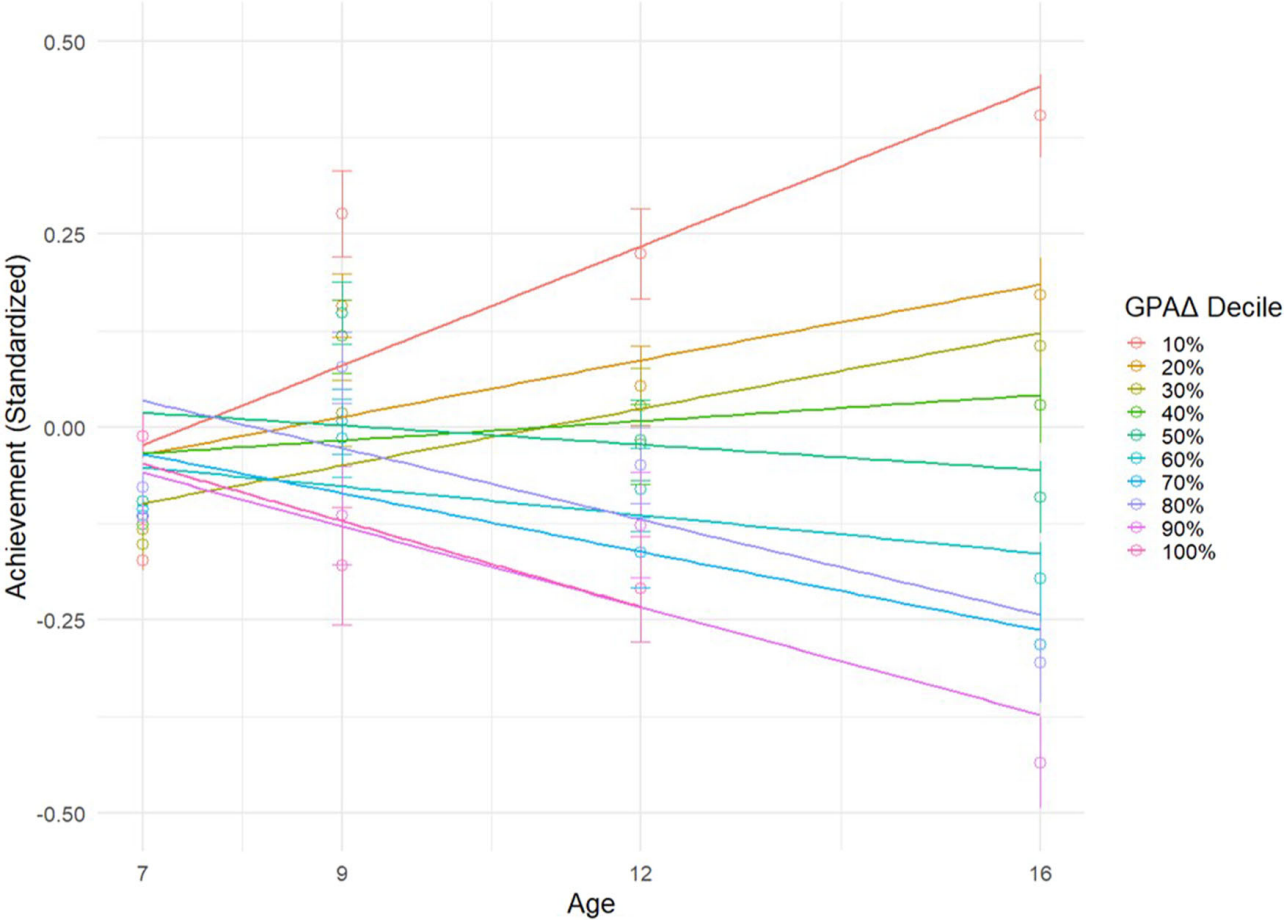
Achievement trajectories by GPAΔ decile. Achievement trajectories among children at ages 7, 9, 12, and 16, grouped by GPAΔ decile (*N* = 1981). This subset contains participants whose achievement scores at age 7 fall within 0.5 standard deviations of the mean. In the graph, individual data points represent the mean achievement scores for each GPAΔ decile group at the specified ages. The lines drawn through these points represent the linear regression lines of best fit for each group.

To investigate whether our sample is regressing towards their genomic predictions, as opposed to the achievement mean, we also examined the trajectories of a subset of children whose achievement was average at age 7 (within 0.5 SD of the mean). We then stratified these children by GPAΔ deciles. For example, the lowest decile of GPAΔ underachievers at age 7 improved by nearly half a standard deviation by age 16 (top line in Fig. 5). Conversely, the achievement of the highest decile of GPAΔ overachievers at age 7 declined by almost half a standard deviation by age 16. See Supplementary Table 3 for means and standard errors by decile.

We then predicted achievement slopes across ages 7, 9, 12, and 16 from GPAΔ at age 7, controlling for sex, age, and family socio-economic status (SES). Although sex and age correlate little with achievement at age 7 (−0.03 and 0.10 respectively), SES correlates 0.30. To determine the unique contribution of GPAΔ, we performed a hierarchical regression where the initial model only included GPAΔ as a predictor (Model 1) and subsequent models added sex, age, and family SES as covariates (see Supplementary Table 4). The unique variance in achievement slopes explained by GPAΔ (or the partial ${R}^{2}$) dropped from 20.8% to 18.5% with the addition of covariates. To compute the significance of this change, we conducted an F-test comparing the residual sum of squares of Model 1 and a full model with all covariates included, which indicated that the full model explained significantly more variance overall (Δ${R}^{2}$ = 0.038; *F*(3, 3926) = 66.949, *p* < 0.001). However, the minimal changes in ${R}^{2}$ suggest that sex, age and family SES do not substantially mediate the relationship between GPAΔ and achievement slopes (Supplementary Table 4). We also found SES did not moderate the relationship between GPAΔ and achievement slope (β interaction term = −0.021, ${\text{CI}}_{95 \% }$ [−0.043, 0.000], *p* = 0.134), suggesting that the relationship between GPAΔ scores and achievement trajectories remains stable across varying SES levels (Supplementary Table 5). These findings indicate that low SES does not prevent underachievers at age 7 from reaching their genomically-predicted academic achievement nor does high SES stop the decline in achievement for GPAΔ overachievers at age 7.

In addition to these analyses based on EA4 GPS, we also explored the use of a multi-polygenic score approach to index predicted achievement. To do this, we ran a 10-fold cross-validation with three repeats in multiple regressions using five GPS related to cognition (including EA4) to predict age 7 achievement (Supplementary Table 6). To test for multicollinearity between predictors, we computed the variance inflation factor (VIF), which estimates how much the variance of a coefficient is inflated due to collinearity with other predictors (see Methods)^28^. All predictors of the final model had VIF values between 1.56 and 4.33, which is below the conventional threshold of 5 (which is used to diagnose if multicollinearity is high)^29^. Thus, updated GPAΔ were then computed based on a weighted mean of all five polygenic scores, using parameter estimates from the cross-validation model, and we compared this multi-GPS GPAΔ against the EA4-only GPAΔ. We found that the multi-GPS model predicted achievement no better than the EA4 GPS alone. The zero-order correlation between the multi-GPS GPAΔ and achievement slopes was −0.430 (${\text{CI}}_{95 \% }$ −0.453, −0.404, *p* < 0.001) as compared to −0.456 for EA4 alone (${\text{CI}}_{95 \% }$-0.480, −0.432], *p* < 0.001), with overlapping confidence intervals. This is likely because prediction was optimized for achievement at age 7, compared to educational attainment, which captures factors related to both childhood and later age achievement. For this reason, we did not use the multi-GPS model in subsequent analyses.

### Associations between GPAΔ scores and achievement slopes separately for English and mathematics

The previous analyses used combined scores in English, mathematics, and science to create achievement slopes. Here, we consider English and mathematics individually. We created achievement slopes separately for English and its associated GPAΔ (English - GPS) and separately for mathematics and its associated GPAΔ (mathematics – GPS) (Supplementary Tables 7 and 8). Science was not assessed in the National Curriculum at age 7.

**Fig. 6:**
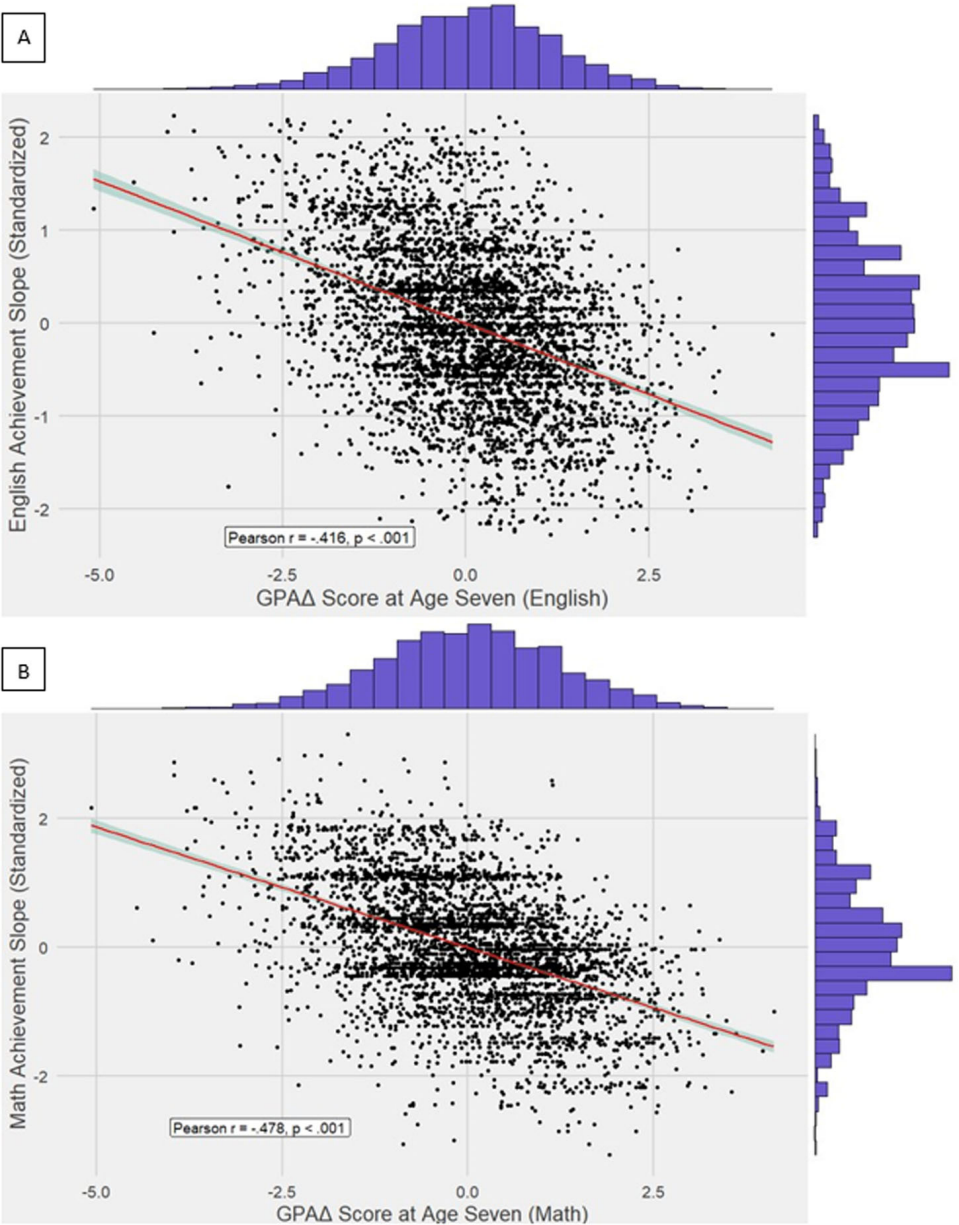
Scatterplot for domain-specific GPAΔ and achievement slopes. Scatterplots separately for English **A** and mathematics **B** GPAΔ scores and achievement slopes.

In both instances, we continued using the EA4 GPS to index ability. Figure 6 shows scatterplots between domain-specific underachievement and their respective achievement slopes. The magnitude of the zero-order correlations was similar to those for overall achievement. The correlations were also similar for English and mathematics but significantly lower for English [*r* = −0.416, (${\text{CI}}_{95 \% }$[−0.440, −0.390], *p* < 0.001)] than for mathematics [*r* = −0.478, (${\text{CI}}_{95 \% }$[−0.501, −0.454], *p* < 0.001)].

### Comparison with general cognitive ability (*g*)

How do these findings for GPAΔ at age 7 compare to those for the traditional index based on the discrepancy between achievement and g (cogΔ; achievement – g)? We found that cogΔ at age 7 correlated 0.377 with GPAΔ (${\text{CI}}_{95 \% }$[−0.345, −0.408], *p* < 0.001), which suggests that results for the two measures could differ. The first difference we observed was their heritability estimates. In contrast to the strikingly high heritability of 85% for GPAΔ, the heritability of cogΔ was only 34.3% (95% CI: 0.240−0.444; Supplementary Table 1).

Contrary to our hypothesis, our genomic index, GPAΔ at age 7, predicted as much of the variance in achievement slopes as cogΔ at age 7. For GPAΔ, the semi-partial ${R}^{2}$ was 0.185 (${\text{CI}}_{95 \% }$[0.164, 0.206], *p* < 0.001) after adjustment for SES, sex, and age. For cogΔ, the semi-partial ${R}^{2}$ was 0.145$\,({\text{CI}}_{95 \% }$[0.122, 0.169], *p* < 0.001). The confidence intervals between the two ${R}^{2}$ were overlapping, indicating that they do not differ significantly. Both regressions were directionally similar, suggesting that individuals regressed to their predicted levels of achievement for both GPAΔ and cog Δ.

The correlation of 0.377 between GPAΔ and cogΔ suggests that they could add independently to the prediction of achievement slopes. We tested their complementarity in a multiple regression of the two Δ measures as they predict achievement slopes and found that together the measures predicted substantially more variance in achievement slopes: ${R}^{2}=\,0.261$, as compared to 0.185 for GPAΔ and 0.145 for cogΔ. In addition to their increased joint prediction, both Δ measures significantly predicted achievement slopes independent of one another. The standardized beta for GPAΔ was −0.351 and the beta for cogΔ was −0.262. Full regression results are included in Supplementary Table 9 and descriptive statistics are shown in Supplementary Table 10.

## Discussion

In the present study, we found that students’ GPAΔ at age 7 was associated with their longitudinal achievement trajectories. GPAΔ underachievers, aligning with their genomic predictions, showed an increase in achievement over time, while GPAΔ overachievers experienced a decrease in achievement over time. For instance, absolute GPA underachievers at age 7 had an average grade of -2 SD at age 7 but by age 16 had achieved an average grade of -1.42 SD—a half standard deviation increase. We acknowledge that these patterns are to be expected theoretically on the basis of regression to the mean, as could any attempt to assess underachievement as a shortfall of achievement in relation to predicted achievement such as discrepancies between achievement and ability. Our goal was to chart empirically the course of trajectories of achievement across the school years. We found that underachievers regress 31% back to their genomically predicted trajectory by the end of compulsory schooling. We found that GPAΔ predicts an equal amount of variance in achievement slopes compared to cogΔ, across both general and domain-specific achievement. Surprisingly, the two Δ are complementary, jointly predicting 26% of the variance in achievement slopes.

These findings suggest that GPAΔ can help identify underachievers in the early school years, with the rationale of maximizing their achievement by personalizing their education. We emphasize *their* achievement because the goal is for each student to achieve their potential, not the unrealistic goal that all children achieve the highest level of performance. As noted earlier, targeting GPAΔ as an early-warning system seems likely to be efficacious. In other words, targeting students underachieving genomically might be more effective than attempting to improve performance of all children. However, we reiterate that this a hypothesis that needs to be tested in intervention research investigating whether GPAΔ underachievers respond better to the intervention than other underachievers. Along these same lines, interventions that are effective in boosting the performance of GPAΔ underachievers might be less effective in preventing the decline of GPAΔ overachievers. Again, this is a hypothesis that needs to be tested in intervention research, which could lead to interventions designed specifically for GPAΔ overachievers.

At present, GPAΔ at age 7 could only be used to identify underachievers and overachievers in the early school years once their achievement has been assessed. We propose, however, that if a GWA study of school achievement were to be conducted (implying the availability of genotypes and school achievement data), then it would be possible to perform a GWA analysis of GPAΔ as well as school achievement per se. A GPS derived from a GWA study of GPAΔ could be created early in life before school achievement is assessed, which would enable early intervention to prevent GPAΔ underachievement and to maintain GPAΔ overachievement before children start school. Our finding of extremely high heritability (85%) for GPAΔ should encourage GWA analyses of GPAΔ.

When considering the finding that school achievement increasingly resembles genomic predictions with age, there are several ways this result can be interpreted. One possibility is that the influence of genetics increases with age. However, this seems unlikely because the heritability of educational achievement does not increase with development^30^ (see Supplementary Table 1). The variance explained by GPS, however, does increase with age, likely because achievement measures at later ages increasingly approximate the target phenotype of the EA4 GWA study, educational attainment, which can only be assessed in adulthood. GCSEs are more strongly correlated with EA4 compared to earlier achievement scores at ages 7, 9, and 12 because they are administered at the end of compulsory schooling. Therefore, one possibility for why students increasingly resemble their genomic predictions is that achievement measures are more correlated with EA4 at later ages. In the case of cogΔ, we also find that students’ achievement regresses towards levels predicted by their intelligence scores at age 7. Intelligence at age 7 correlates at 42 with intelligence at age 16^7^, while EA4 correlates around 0.40 with achievement at age 16, suggesting both should be equally predictive of ability at age 16.

Indeed, this is what we find: GPAΔ and cogΔ are equally predictive of achievement slopes, despite being correlated 0.4 with one another. The imperfect correlation between the two Δ measures could be attributed to their measurement of distinct (but complementary) aspects of school ability. GPAΔ, represented by a GPS of educational attainment, encompasses cognitive and school-specific non-cognitive effects, whereas g solely captures cognitive abilities. We find that, jointly, the two measures predict more variance in achievement slopes (26%) than GPAΔ (19%) or cogΔ (15%) alone.

For both GPAΔ and cogΔ, the range of individual differences in trajectories through the school years should be noted—about 85% of the variance in achievement slopes is not explained by either index. Interestingly, GPAΔ was able to predict achievement slopes in general achievement, English, and math to a similar degree. This finding illustrates the generality of educational attainment assessed by the EA4 GPS, despite being a measure downstream from school achievement. We are extending the present work to consider the antecedents and sequalae of individual differences in GPAΔ.

Our study also found that SES does not moderate the relationship between achievement slopes and GPAΔ, suggesting that underachieving students from low-SES backgrounds are not any slower in returning to their genomically predicted levels of achievement compared to their high-SES counterparts. Conversely, high SES does not act as a protective factor in preventing overachievers from experiencing declines in their achievement. However, it is worth mentioning that SES is higher in the relative underachievers group and lower in the relative overachievers group. One possibility is that high family SES may alleviate the pressure for students to do well in school, making them believe they will progress to higher education regardless, or have parental support to fall back on^31^. We refrain from discussing the role of SES in underachievement and overachievement, as this and other explanations would be speculative.

There are several limitations to our study. Given that we examine longitudinal data across nine years, it is unsurprising that some participants are lost due to attrition. To maximize sample size, we selected participants who had observations across any 3 out of the possible 4 years. Other limitations include our inability to control for classroom and school effects and our use of teacher assessments. However, classroom and school effects are surprisingly small after taking SES into account and our teacher assessments are based on National Curriculum rating guidelines and correlate highly with standardised test scores (see Methods).

A general limitation of genomic research concerns ethnicity. Although we argue that GPS provide an unbiased estimate of ability with regards to training and practice effects, they do not evade the clutches of cultural bias. GWA studies have largely included northern European ancestries and the predictive power of GPS is attenuated in populations with different ancestries^32,33^. In our study, we included only those who identified themselves as having European ancestry meaning our results likely represent the upper-bound of prediction for other ancestries. While the ethical issues of using genomics for prediction has been extensively covered^34,35^, our focus in the present study is on the scientific and practical aspects of predicting underachievement.

The current study explored empirically the extent to which underachievement and overachievement could usefully be defined using genomic data. To accomplish this, we devised an index called Genomically-Predicted Achievement Discrepancy (GPAΔ) by subtracting a GPS for adult educational attainment from educational achievement at age 7. We demonstrated that students who exhibited achievement-GPS discrepancies at age 7 on average regressed a third of the way back to their genomically predicted levels of achievement at ages 9, 12, and 16.

## Methods

### Sample

At its conception, TEDS recruited over 16,000 twin pairs born between 1994-1996 using national birth records (Rimfeld et al.^36^). Ethical approval for this study was received from King’s College London Ethics Committee. The present study used a sub-sample of twins who contributed to data collection between 7–16 years of age. Namely, we used data collected during four waves, which correspond to the four key stages of education in the UK: 7, 9, 12, and 16. Although the total sample size varies at each wave due to attrition and budgetary constraints (for example, at age 9 only two of four yearly cohorts could be assessed), the sample continues to remain representative of the UK general population^36^. We genotyped up to 10,346 individual participants: 2666 MZ twins (one member per twin pair was genotyped), 4337 DZ twins (one member per twin pair), 3320 DZ co-twins, and 23 twins of unknown zygosity. Of the available participants, we included those who self-identified as having European ancestry, and excluded those who reported having a serious medical condition, leaving a sample size of *N* = 9694. Lastly, to capture longitudinal trajectories, we stipulated that all participants needed a minimum of three achievement observations across any three of the four ages, resulting in a final sample size of *N* = 4175.

### Genotyping

DNA for 12,500 individuals in the TEDS sample was extracted from saliva and buccal cheek swab samples and hybridized to one of two SNP microarrays (Affymetrix GeneChip 6.0 or Illumina HumanOmniExpressExome chips). Standard quality control (QC) steps were undertaken to remove low quality samples: samples were removed from subsequent analyses on the basis of call rate (<0.98), suspected non-European ancestry, heterozygosity, and relatedness other than dizygotic twin status. SNPs were excluded if the minor allele frequency was smaller than 0.5%, if more than 2% of genotype data were missing, or if the Hardy Weinberg p-value was lower than ${10}^{-5}$. Non-autosomal markers and indels were removed. Association between SNP and the platform, batch, plate or well on which samples were genotyped was calculated; SNPs with an effect p-value < ${10}^{-4}$ were excluded. A total sample size of 10,346 (including 7026 unrelated individuals and 3320 additional dizygotic co-twins) remained after QC. For detailed description of subsequent phasing, imputation, and genomic principal component generation, see ref.^37^.

### Measures

- **National Curriculum levels at ages 7, 9, and 12:** We used UK National Curriculum (NC) teacher-assessed grades at ages 7, 9, and 12. These marks are based on externally provided tests and tasks provided by the National Foundation for Educational Research (NFER) and the Qualifications and Curriculum Authority (QCA) (NFER: http://www.nfer.ac.uk/index.cfm; QCA: http://www.qca.org.uk), which adhere to a standardized UK National Curriculum rubric. At age 7 and 9 the curriculum is rated on a 5-point scale, and at age 12 on a 9-point scale, with higher values representing higher achievement. NC data and genotypes were available for age 7 (M = 2.14, SD = 0.52, *N* = 6740), age 9 (M = 3.01, SD = 0.58, *N* = 3206), and age 12 (M = 4.41, SD = 0.89, *N* = 4554). Achievement data were available for English, math, and science. We standardized these scores and computed their mean to establish a composite measure of general achievement. At age 7, the composite was computed using English and math, as science data were not available. Subsequent data obtained from the National Pupil Database (NPD) show that teacher-rated assessments correlated *r* = 0.80 with exam scores, while GCSE grades collected by TEDS correlated between > 0.95 and 0.99 with NPD records^38^.
- **General Certificate of Secondary Education at age 16:** The General Certificate of Secondary Education (GCSE) is a standardized examination taken at the end of compulsory schooling, usually at age 16. In the present study, we used the core subjects, consisting of English, science, and math, to construct achievement composites. The grades system ranges from 4 (G; minimum pass grade) to 11 (A*; highest possible grade). We standardized these scores and computed their mean to establish a composite measure of general achievement, analogous to our NC measures. GCSE data was available for 7347 genotyped participants (M = 8.92, SD = 1.22).
- **General Cognitive Ability at age 7:** To measure general cognitive ability, twins were assessed via telephone on four tests: Conceptual Grouping, Similarities, Vocabulary and Picture Completion^39,40^. We standardized these scores and computed their mean to establish a composite measure of general cognitive ability (*g*). These telephone-based assessments have been shown to correlate with in-person assessments, reaching *r* = 0.72 after range correction (Petrill et al.^41^).
- **Family SES:** At first contact, parents of the TEDS participants completed a questionnaire by post, which included questions about mother and father employment levels, mother and father educational levels, and mother’s age on birth of first child. SES was calculated using a mean of the five variables, and standardized. Data was available for 9156 genotyped participants.

### Statistical analyses

The study was preregistered (https://osf.io/xhb2s/) in relation to the design, methods, measures and general analyses and hypotheses. Although our results are consistent with the hypotheses, our analyses that we report here went well beyond our initial plans and therefore we are choosing to report our findings as exploratory rather than confirmatory.

#### Outlier removal

We used the median absolute deviation (MAD) method for outlier removal of achievement slope scores, as this approach is robust against deviations from normality^42^. Scores exceeding three times the MAD were considered outliers and removed. MAD is defined in Eq. (1).

#### Genome-wide polygenic scores (GPS)

We employed LDPred2, a Bayesian method that corrects for local linkage disequilibrium, to compute GPS for all genotyped participants. For an in-depth explanation of GPS construction in TEDS, see^37^. All GPS were residualized against chip type, batch, and their first ten principal components. Our core analyses were done using summary statistics sourced from EA4, the largest GWA study of educational attainment (Okbay et al.^13^). Due to data access restrictions, the EA4 summary statistics used in this study have been computed without 23andMe participants, with a sample size of *N* = 765,283. Thus, the participants used to compute the summary statistics for EA4 have nearly identical participant overlap with the previous GWAS of educational attainment, EA3^15^. For our multi-polygenic score analyses, we also made use of GPS for IQ^14^, cognitive performance^15^, self-reported math ability^15^, and math attainment^15^.

#### Identifying Underachievement

Identifying academic underachievement and overachievement is challenging due to variability in identification methods. These methods often lack agreement, suggesting that distinct populations might be labeled as underachievers by different researchers (Jackson & Jung^43^; White et al.^44^). The most conservative approach is to ensure that ability-achievement discrepancies are sufficiently large to rule out measurement error. This is done, for example, by requiring underachievers to surpass a certain ability threshold (+1 SD) while falling below another achievement threshold (−1 SD). However, this approach tends to disproportionately identify gifted individuals as underachievers and fails to consider those with average or even below-average abilities^45^. Furthermore, by dichotomizing underachievement, it is unable to distinguish the *extent* of one’s underachievement, which is a continuum^4^.

To overcome these issues, we employ a difference-score approach in which standardized predicted achievement, as indexed by a GPS, is subtracted from standardized observed achievement. This results in a continuous scale representing a spectrum from underachievement to overachievement. Under this model, negative scores indicate underachievement, that is, the student’s GPS exceeds their achievement. Conversely, positive values indicate *overachievement*, which refers to achievement exceeding expectations. We wanted to challenge prior conceptions by incorporating both underachievers and overachievers under a general phenotype indexing individual differences in achievement-ability discrepancies throughout the continuum, as well as considering the underachieving and overachieving extremes of the distribution. Our rationale for using a difference-score approach rather than residualizing achievement for GPS (as is also commonly done) is described previously. However, residualized scores yield similar results (Supplementary Table 11 and Supplementary Figs. 1 and 2).

#### Multi-polygenic score analyses

For our multiple polygenic score analyses^16^, we ran a 10-fold cross-validation with three repeats in multiple regressions using the five previously mentioned GPS related to cognition (including EA4) to predict age 7 achievement using the *caret* R package^46^. We trained our model to maximize prediction at age 7 achievement, because GPS is being used as a proxy for ability/expected achievement at that age. When comparing correlations, we used a confidence interval procedure to estimate the direction and magnitude of the effect, with the *cocor* R package^47,48^. To test for multicollinearity in the final cross-validation model, we computed the variance inflation factor for all predictors using the *car* R package^49^.

#### Twin design

The twin design was used to estimate univariate heritability across GPAΔ and related traits. Twins offer a natural experiment in disentangling the effects of genes and environment through our a priori knowledge of their genetic similarity: MZ twins share 100% of their genes, while DZ twins on average share 50% of their genes. Using this assumption, alongside several others such as the equal environment assumption (which states that MZ and DZ twins are exposed to shared environments to similar degrees), it is possible to estimate the effects of additive genetic (A), shared environmental (C), and non-shared environmental (E) variance on phenotypic variance using model-fitting approaches^10^. We used the R package *OpenMx* for all model-fitting analyses^50^.

### Reporting summary

Further information on research design is available in the Nature Research Reporting Summary linked to this article.

### Supplementary information

Supplemental Material

reporting summary

## Data Availability

The data used in this study come from the Twins Early Development Study (TEDS). Researchers can apply for access to TEDS data for academic purposes through the creation of a Data Access agreement. The full data access policy can be found at the following link: https://www.teds.ac.uk/researchers/teds-data-access-policy.

## Code availability

The corresponding author will share the code upon request.

## References

[1] Badger JR, Howarth BC, Svirko E, Mellanby J (2022). Underachievement at school relative to potential: links between reasoning, phonological decoding, short-term memory, and complex grammar. Educ. Psychol..

[2] Heckman JJ (2006). Skill formation and the economics of investing in disadvantaged children. Science.

[3] Vignoles A, Murray N (2016). Widening participation in higher education. Educ. Sci..

[4] Preckel F, Holling H, Vock M (2006). Academic underachievement: Relationship with cognitive motivation, achievement motivation, and conscientiousness. Psychol. Sch..

[5] Reis SM, McCoach DB (2000). The underachievement of gifted students: what do we know and where do we go? *Gift*. Child Q.

[6] Davey, G. C. *Psychology*. (John Wiley & Sons, 2018).

[7] Von Stumm S, Plomin R (2015). Socioeconomic status and the growth of intelligence from infancy through adolescence. Intelligence.

[8] Plomin, R. *Blueprint: How DNA Makes Us Who We Are*. (Penguin Books, London, 2019).

[9] Visscher PM (2017). 10 years of GWAS discovery: biology, function, and translation. Am. J. Hum. Genet..

[10] Knopik, V. S., Neiderhiser, J. M., DeFries, J. C. & Plomin, R. *Behavioral Genetics*. (Worth Publishers, Macmillan Learning New York, 2017).

[11] Fahed AC, Philippakis AA, Khera AV (2022). The potential of polygenic scores to improve cost and efficiency of clinical trials. Nat. Commun..

[12] Allegrini AG (2019). Genomic prediction of cognitive traits in childhood and adolescence. Mol. Psychiatry.

[13] Okbay A (2022). Polygenic prediction of educational attainment within and between families from genome-wide association analyses in 3 million individuals. Nat. Genet..

[14] Savage JE (2018). Genome-wide association meta-analysis in 269,867 individuals identifies new genetic and functional links to intelligence. Nat. Genet..

[15] Lee JJ (2018). Gene discovery and polygenic prediction from a genome-wide association study of educational attainment in 1.1 million individuals. Nat. Genet..

[16] Krapohl E (2018). Multi-polygenic score approach to trait prediction. Mol. Psychiatry.

[17] Procopio, F. et al. *Multi-Polygenic Score Prediction of Mathematics, Reading, and Language Abilities Independent of General Cognitive Ability*. https://www.researchsquare.com/article/rs-3573692/v1 (2024) 10.21203/rs.3.rs-3573692/v1. 10.1038/s41380-024-02671-w PMC11746139 39085392

[18] Snyder KE (2019). Interventions for academically underachieving students: A systematic review and meta-analysis. Edu. Res. Rev..

[19] Steenbergen-Hu S, Olszewski-Kubilius P, Calvert E (2020). The effectiveness of current interventions to reverse the underachievement of gifted students: findings of a meta-analysis and systematic review. Gift. Child Q..

[20] Majumder MA, Guerrini CJ, McGuire AL (2021). Direct-to-consumer genetic testing: value and risk. Annu. Rev. Med..

[21] Niemiec E, Kalokairinou L, Howard HC (2017). Current ethical and legal issues in health-related direct-to-consumer genetic testing. Pers. Med..

[22] Abdellaoui A, Yengo L, Verweij KJH, Visscher PM (2023). 15 years of GWAS discovery: realizing the promise. Am. J. Hum. Genet..

[23] Baker JA, Bridger R, Evans K (1998). Models of underachievement among gifted preadolescents: the role of personal, family, and school factors. Gift. Child Q..

[24] Snyder KE, Linnenbrink-Garcia L (2013). A developmental, person-centered approach to exploring multiple motivational pathways in gifted underachievement. Educ. Psychol..

[25] Peterson JS, Colangelo N (1996). Gifted achievers and underachievers: a comparison of patterns found in school files. J. Couns. Dev..

[26] Carr, M., Borkowski, J. G. & Maxwell, S. E. Motivational Components of Underachievement. (1991).

[27] Vlahovic‐Stetic V, Vidovic VV, Arambasic L (1999). Motivational Characteristics in Mathematical Achievement: a study of gifted high‐achieving, gifted underachieving and non‐gifted pupils. High. Abil. Stud..

[28] Thompson CG, Kim RS, Aloe AM, Becker BJ (2017). Extracting the variance inflation factor and other multicollinearity diagnostics from typical regression results. Basic Appl. Soc. Psychol..

[29] Salmerón R, García CB, García J (2018). Variance inflation factor and condition number in multiple linear regression. J. Stat. Comput. Simul..

[30] Kovas Y (2013). Literacy and numeracy are more heritable than intelligence in primary school. Psychol. Sci..

[31] Haider, Z. F. & von Stumm, S. Predicting educational and social–emotional outcomes in emerging adulthood from intelligence, personality, and socioeconomic status. *J. Pers. Soc. Psychol*. (2022). 10.1037/pspp0000421 35549304

[32] Duncan L (2019). Analysis of polygenic risk score usage and performance in diverse human populations. Nat. Commun..

[33] Martin AR (2019). Clinical use of current polygenic risk scores may exacerbate health disparities. Nat. Genet..

[34] Lewis ACF, Green RC (2021). Polygenic risk scores in the clinic: new perspectives needed on familiar ethical issues. Genome Med..

[35] Palk AC, Dalvie S, De Vries J, Martin AR, Stein DJ (2019). Potential use of clinical polygenic risk scores in psychiatry – ethical implications and communicating high polygenic risk. Philos. Ethics Humanit. Med..

[36] Rimfeld K (2019). Twins early development study: a genetically sensitive investigation into behavioral and cognitive development from infancy to emerging adulthood. Twin Res. Hum. Genet..

[37] Selzam S (2017). Predicting educational achievement from DNA. Mol. Psychiatry.

[38] Rimfeld K (2018). The stability of educational achievement across school years is largely explained by genetic factors. Npj Sci. Learn..

[39] McCarthy, D. A. Manual for the McCarthy scales of children’s abilities. *No Title* (1972).

[40] Wechsler, D. Manual for the wechsler intelligence scale for children-third UK Edition (WISC-III UK). *Kent Psychol. Corp*. (1992).

[41] Petrill SA, Rempell J, Oliver B, Plomin R (2002). Testing cognitive abilities by telephone in a sample of 6- to 8-year-olds. Intelligence.

[42] Leys C, Ley C, Klein O, Bernard P, Licata L (2013). Detecting outliers: do not use standard deviation around the mean, use absolute deviation around the median. J. Exp. Soc. Psychol..

[43] Jackson RL, Jung JY (2022). The identification of gifted underachievement: validity evidence for the commonly used methods. Br. J. Educ. Psychol..

[44] White SLJ, Graham LJ, Blaas S (2018). Why do we know so little about the factors associated with gifted underachievement? A systematic literature review. Educ. Res. Rev..

[45] Fong, C. J. & Kremer, K. P. An expectancy-value approach to math underachievement: examining high school achievement, college attendance, and STEM Interest. *Gift. Child Q*. (2020).

[46] Kuhn, M. Building Predictive Models in *R* Using the caret Package. *J. Stat. Softw*. **28**, (2008).

[47] Diedenhofen B, Musch J (2015). cocor: a comprehensive solution for the statistical comparison of correlations. PLoS ONE.

[48] Zou GY (2007). Toward using confidence intervals to compare correlations. Psychol. Methods.

[49] Fox J (2012). Package ‘car’. Vienna R. Found. Stat. Comput..

[50] Neale MC (2016). OpenMx 2.0: extended structural equation and statistical modeling. Psychometrika.

