# Supplementary material for: Exploring the genetic prediction of academic underachievement and overachievement
Source: NPJ Sci Learn. 2024 Jun 1;9:39. doi: 10.1038/s41539-024-00251-9 (PMC11144217; doi:10.1038/s41539-024-00251-9)

Supplementary Tables and Figures For

Exploring the genetic prediction of academic underachievement and overachievement

Contents

Supplementary Table 1: Mean and Standard Deviations for measured variables across groups 3
Supplementary Table 2: Twin Intra-class correlations and ACE Estimates..... 4
Supplementary Table 3: Means and Standard Errors by GPAΔ Decile **5-7**
Supplementary Table 4: Multiple Regression results using slope as the predicted variable..... 8
Supplementary Table 5: Regression results from moderation by SES 9
Supplementary Table 6: Multi-GPS Prediction..... **10**
Supplementary Table 7: Regression outputs for Domain-Specific Achievement (English)..... **11**
Supplementary Table 8: Regression outputs for Domain-Specific Achievement (mathematics) **12**
Supplementary Table 9: Regression outputs for cogΔ..... **13**
Supplementary Table 10: Descriptive statistics for English and mathematics **14**
Supplementary Table 11: GPAΔ using Residualized Scores..... **15**
Supplementary Table 12: Pairwise correlations between all measured variables..... **16-19**
Supplementary Figure 1: Scatterplot between GPAΔ (residualized) and Slope..... **20**
Supplementary Figure 2 Scatterplot between GPAΔ (residualized) and GPAΔ (difference score) **21**

Supplementary Table 1: Mean (SD) for measured variables across groups (unstandardized unless indicated otherwise). Note: Standardization performed prior to application of exclusion criteria.

	Whole Sample (*N* = 4175)	Relative Underachievers (*N* = 754)	Relative Overachievers (*N* = 857)	Absolute Underachievers (*N* = 213)	Absolute Overachievers (*N* = 405)
Age 7 Composite Achievement	2.21 (0.5)	1.79 (0.52)	2.58 (0.40)	1.05 (0.36)	2.94 (0.11)
Age 9 Composite Achievement	3.08 (0.56)	2.85 (0.61)	3.26 (0.54)	2.20 (0.53)	3.53 (0.42)
Age 12 Composite Achievement	4.5 (1.19)	4.24 (1.02)	4.65 (0.82)	3.31 (0.90)	5.00 (0.86)
Age 16 Composite Achievement	8.99 (1.19)	8.70 (1.42)	9.12 (1.13)	7.27 (1.29)	9.76 (0.90)
Achievement Slopes (standardized)	0 (0.93)	0.57 (0.85)	-0.07 (0.08)	0.07 (0.10)	-0.09 (0.08)
EA4 Polygenic Score (standardized)	0.1 (0.99)	-0.18 (0.34)	-0.86 (0.29)	-0.57 (0.32)	-0.63 (0.30)
SES (standardized)	0.28 (0.93)	0.38 (0.99)	0.11 (0.89)	-0.18 (0.95)	0.45 (0.93)
Sex	2273 males	373 males	462 males	90 males	214 males
Age when sitting GCSEs	16.31 (0.28)	16.26 (0.29)	16.34 (0.28)	16.24 (0.29)	16.24 (0.29)

Supplementary Table 2: Twin Intra-class correlations (95% CI) and model fitting results for univariate twin analyses, given as additive genetic (A), shared environmental (C), and non-shared environmental (E) with 95% CI in parentheses. All variance components have been standardized.

	**Twin Correlations**		**Variance Components**		
Trait	**rMZ**	**rDZ**	**A**	**C**	**E**
**Age 7 Achievement**	.857 (.839 – 872), N = 980	.521 (.494 – 548), N = 2803	.674 (.619 - .731)	.184 (.130 - .236)	.141 (.129 - .156)
**Age 7 English**	.839 (.819 - .857), N = 975	.496 (.468 – 534), N = 2787	.679 (.621 - .739)	.158 (.102 - .211)	.163 (.148 – .179)
**Age 7 Math**	.812 (.789 - .832), N = 974	.467 (.438 - .496), N = 2784	.706 (.644 - .769)	.112 (.053 - .168)	.182 (.166 - .200)
**Age 9 Achievement**	.838 (.811 - .861), N = 545	.494 (.455 - .531), N = 1516	.736 (.655 - .801)	.117 (.037 - .192)	.148 (.130 – 169)
**Age 12 Achievement**	.861 (.840 - .880), N = 685	.621 (.592 - .648), N = 1845	.500 (.444 - .558)	.374 (.319 - .426)	.126 (.112 - .142)
**Age 16 Achievement**	.888 (.874 - .900), N = 990	.564 (.538 – 589), N = 2837	.663 (.613 - .715)	.229 (.179 - .278)	.108 (.098 - .119)
**Achievement Slopes**	.781 (.754 - .804), N = 1019	.470 (.441 - .497), N = 2898	.565 (.485 - .646)	.203 (.129 – 276)	.232 (.210 – 258)
**GPAΔ**	.911 (.900 - .921), N = 980	.485 (.442 - .526), N = 1251	.851 (.773 - .935)	.060 (.000 - .141)	.090 (.082 - .100)
**CogΔ**	.623 (.576 - .667), N = 692	.451 (.414 - .486), N = 1902	.343 (.240 - .444)	.277 (.199 - .354)	.379 (.341 - .423)
**English GPAΔ**	.887 (.874 - .899), N = 1061	.458 (.413 - .501), N = 1248	.845 (.763 - .934)	.040 (.000 - .123)	.115 (.106 - .126)
**Math GPAΔ**	.897 (.884 – 908), N = 1056	.457 (.411 - .500), N = 1216	.870 (.788 - .960)	.026 (.000 - .109)	.105 (.097 – .115)

Supplementary Table 3: Means and Standard Errors of Achievement by GPAΔ Decile

Age	GPAΔ Decile	Mean Achievement (standardized)	SE Mean	*N*
7	10%	-0.172	0.014	195
7	20%	-0.133	0.013	272
7	30%	-0.153	0.013	295
7	40%	-0.126	0.014	266
7	50%	-0.097	0.015	280
7	60%	-0.115	0.014	263
7	70%	-0.106	0.016	230
7	80%	-0.078	0.017	221
7	90%	-0.117	0.018	173
7	100%	-0.012	0.029	95
9	10%	0.277	0.056	195
9	20%	0.157	0.041	272
9	30%	0.018	0.042	295
9	40%	0.117	0.048	266
9	50%	0.148	0.040	280

Age	GPAΔ Decile	Mean Achievement (standardized)	SE Mean	*N*
9	60%	-0.014	0.050	263
9	70%	0.007	0.042	230
9	80%	0.077	0.046	221
9	90%	-0.114	0.063	173
9	100%	-0.180	0.076	95
12	10%	0.225	0.059	195
12	20%	0.053	0.051	272
12	30%	0.027	0.049	295
12	40%	-0.022	0.051	266
12	50%	-0.018	0.052	280
12	60%	-0.081	0.054	263
12	70%	-0.163	0.046	230
12	80%	-0.049	0.049	221
12	90%	-0.127	0.068	173
12	100%	-0.209	0.068	95
16	10%	0.403	0.054	195

Age	GPAΔ Decile	Mean Achievement (standardized)	SE Mean	*N*
16	20%	0.172	0.048	272
16	30%	0.106	0.042	295
16	40%	0.029	0.050	266
16	50%	-0.091	0.047	280
16	60%	-0.196	0.046	263
16	70%	-0.282	0.053	230
16	80%	-0.305	0.052	221
16	90%	-0.435	0.059	173
16	100%	-0.510	0.081	95

Supplementary Table 4: Multiple regression results using slope as the predicted variable.

	Dependent variable: Slope							
	Unstandardized (1)	Standardized (1)	Unstandardized (2)	Standardized (2)	Unstandardized (3)	Standardized (3)	Unstandardized (4)	Standardized (4)
GPAΔ	-0.350*** (-0.371, -0.330)	-0.456*** (-0.483, -0.429)	-0.339*** (-0.359, -0.318)	-0.442*** (-0.469, -0.415)	-0.340*** (-0.361, -0.319)	-0.444*** (-0.471, -0.417)	-0.336*** (-0.357, -0.315)	-0.441*** (-0.468, -0.413)
SES			0.155*** (0.129, 0.181)	0.162*** (0.135, 0.190)	0.154*** (0.129, 0.180)	0.162*** (0.135, 0.189)	0.165*** (0.139, 0.191)	0.174*** (0.147, 0.201)
Sex					-0.124*** (-0.172, -0.076)	-0.069*** (-0.096, -0.042)	-0.122*** (-0.170, -0.073)	-0.068*** (-0.096, -0.041)
Age							-0.170*** (-0.255, -0.085)	-0.054*** (-0.082, -0.027)
Constant	-0.007 (-0.031, 0.018)		-0.045*** (-0.071, -0.020)		0.012 (-0.022, 0.046)		2.774*** (1.388, 4.161)	
Observations	4,175	4,175	4,027	4,027	4,027	4,027	3,931	3,931
R^2	0.208	0.208	0.235	0.235	0.240	0.240	0.252	0.252
Adjusted R^2	0.208	0.208	0.235	0.235	0.239	0.239	0.251	0.251
Residual Std. Error	0.794 (df = 4173)	0.890 (df = 4173)	0.781 (df = 4024)	0.875 (df = 4024)	0.778 (df = 4023)	0.872 (df = 4023)	0.766 (df = 3926)	0.865 (df = 3926)
F Statistic	1,097.856*** (df = 1; 4173)	1,097.856*** (df = 1; 4173)	618.822*** (df = 2; 4024)	618.822*** (df = 2; 4024)	423.537*** (df = 3; 4023)	423.537*** (df = 3; 4023)	330.240*** (df = 4; 3926)	330.240*** (df = 4; 3926)

Note: SES refers to first-contact SES and age refers to age when sitting GCSEs. Sex is coded as 0 = Female, 1 = Male. 95% CI in parentheses. *p<0.1; **p<0.05; ***p<0.01.

Supplementary Table 5: Regression results from moderation by SES using slope as the predicted variable.

	Dependent variable: Slope	
	Unstandardized	Standardized
GPAΔ	-0.335***	-0.437***
	(-0.356, -0.314)	(-0.459, -0.416)
SES	0.154***	0.162***
	(0.128, 0.180)	(0.136, 0.187)
GPAΔ * SES	-0.016	-0.021
	(-0.038, 0.005)	(-0.043, 0.0003)
Constant	-0.047***	
	(-0.072, -0.022)	
Observations	4,027	4,027
R^2	0.236	0.236
Adjusted R^2	0.235	0.235
Residual Std. Error (df = 4023)	0.780	0.780
F Statistic (df = 3; 4023)	413.423***	413.423***

Note: Sex is coded as 0 = Female, 1 = Male. 95% CI in parentheses.
*p<0.1; **p<0.05; ***p<0.01

Supplementary Table 6: Multi-GPS Prediction using age 7 composite achievement (English and Math) as the predicted variable.

Model Fit Index	RMSE	R-Squared	MAE
	0.909	.096	0.689

	Dependent Variable: Age 7 composite achievement	
	Unstandardized	Standardized
Educational Attainment	0.155***	0.161***
	(0.120, 0.189)	(0.127, 0.196)
Intelligence	0.085***	0.089***
	(0.039, 0.131)	(0.043, 0.135)
Self-Reported Math Ability	0.012	0.012
	(-0.043, 0.066)	(-0.042, 0.067)
Highest Math Class Completed	-0.004	-0.004
	(-0.060, 0.053)	(-0.060, 0.053)
Cognitive Performance	0.107***	0.112***
	(0.060, 0.155)	(0.065, 0.159)
Constant	0.088***	
	(0.061, 0.116)	
Observations	4,255	4,255
R^2	0.096	0.096
Adjusted R^2	0.094	0.094
Residual Std. Error (df = 4249)	0.909	0.909
F Statistic (df = 5; 4249)	89.764***	89.764***
Note:		*p<0.1; **p<0.05; ***p<0.01

Supplementary Table 7: Regression outputs for Domain-Specific Achievement using English slope as the predicted variable.

	Dependent variable: English Slope	
	Unstandardized	Standardized
English GPAΔ	-0.306***	-0.416***
	(-0.326, -0.286)	(-0.436, -0.395)
Constant	-0.0003	
	(-0.024, 0.023)	
Observations	4,213	4,213
R^2	0.173	0.173
Adjusted R^2	0.173	0.173
Residual Std. Error (df = 4211)	0.781	0.781
F Statistic (df = 1; 4211)	879.249***	879.249***
Note:		*p<0.1; **p<0.05; ***p<0.01

Supplementary Table 8: Regression outputs for Domain-Specific Achievement using math slope as the predicted variable.

	Dependent variable: Math Slope	
	Unstandardized	Standardized
Math GPAΔ	-0.374***	-0.478***
	(-0.395, -0.353)	(-0.499, -0.457)
Constant	-0.005	
	(-0.030, 0.020)	
Observations	4,246	4,246
R^2	0.228	0.228
Adjusted R^2	0.228	0.228
Residual Std. Error (df = 4244)	0.828	0.828
F Statistic (df = 1; 4244)	1,255.803***	1,255.803***
Note:		*p<0.1; **p<0.05; ***p<0.01

Supplementary Table 9: Regression output for joint prediction using GPAΔ and cogΔ

	Dependent variable: Slope	
	Unstandardized	Standardized
GPAΔ	-0.265***	-0.351***
	(-0.290, -0.239)	(-0.377, -0.326)
cogΔ	-0.228***	-0.262***
	(-0.258, -0.199)	(-0.291, -0.232)
Constant	0.016	
	(-0.012, 0.044)	
Observations	2,884	2,884
R^2	0.261	0.261
Adjusted R^2	0.261	0.261
Residual Std. Error (df = 2881)	0.768	0.768
F Statistic (df = 2; 2881)	508.923***	508.923***
Note:		*p<0.1; **p<0.05; ***p<0.01

Supplementary Table 10: Descriptive statistics for English and mathematics (unstandardized unless indicated otherwise)

English Achievement ($N = 4257$)		
Variable	Mean (SD)	Percentage Missing
English GPAΔ (standardized)	0.01 (1.19)	0.42
English Achievement Slope (standardized)	-0.01 (0.86)	0.00
Age 7 English Achievement	2.21 (0.52)	0.00
Age 9 English Achievement	3.10 (0.63)	49.38
Age 12 English Achievement	4.47 (0.88)	27.23
Age 16 English Achievement	9.00 (1.17)	2.28
SES (standardized)	0.29 (0.93)	3.45

Math Achievement ($N = 4267$)		
Variable	Mean (SD)	Percentage Missing
Math GPAΔ (standardized)	0.01 (1.20)	0.00
Math Achievement Slope (standardized)	-0.01 (0.94)	0.00
Age 7 Math Achievement	2.20 (0.53)	0.07
Age 9 Math Achievement	3.09 (0.63)	48.82
Age 12 Math Achievement	4.45 (0.89)	27.09
Age 16 Math Achievement	8.99 (1.20)	3.75
SES (standardized)	0.28 (0.94)	3.56

Supplementary Table 11: GPAΔ using Residualized Scores

	Dependent variable: Slope	
	Unstandardized	Standardized
Residualized GPAΔ	-0.385***	-0.432***
	(-0.410, -0.361)	(-0.456, -0.407)
Constant	-0.018	
	(-0.043, 0.006)	
Observations	4,175	4,175
R^2	0.186	0.186
Adjusted R^2	0.186	0.186
Residual Std. Error (df = 4173)	0.805	0.805
F Statistic (df = 1; 4173)	956.184***	956.184***
Note:		*p<0.1; **p<0.05; ***p<0.01

Supplementary Table 12: Pairwise correlations between all measured variables

Pearson's Correlations

		N	Pearson's r	*p*	Lower 95% CI	Upper 95% CI
age7edu	- age9edu	2117	0.652	< .001	0.627	0.676
age7edu	- age12edu	2988	0.502	< .001	0.475	0.529
age7edu	- age16edu	4070	0.626	< .001	0.607	0.645
age7edu	- ases	4027	0.304	< .001	0.275	0.331
age7edu	- age	4071	0.103	< .001	0.072	0.133
age7edu	- difference_7	4175	0.567	< .001	0.546	0.587
age7edu	- EA4_no23andme_Okbay2022	4175	0.275	< .001	0.246	0.303
age7edu	- Maths_ability_self_report_Lee2018	4175	0.126	< .001	0.096	0.156
age7edu	- Cognitive_performance_Lee2018	4175	0.267	< .001	0.238	0.295
age7edu	- Maths_highest_class_completed_Lee2018	4175	0.147	< .001	0.117	0.177
age7edu	- IQ_Savage2018_FRCT1	4175	0.256	< .001	0.228	0.284
age7edu	- slope	4175	-0.362	< .001	-0.388	-0.335
age7edu	- g_difference	2884	0.514	< .001	0.487	0.541
age9edu	- age12edu	930	0.621	< .001	0.580	0.659
age9edu	- age16edu	2012	0.648	< .001	0.622	0.672
age9edu	- ases	2052	0.302	< .001	0.262	0.341
age9edu	- pcexgcseage1	2013	0.113	< .001	0.070	0.156
age9edu	- difference_7	2117	0.288	< .001	0.248	0.327
age9edu	- EA4_no23andme_Okbay2022	2117	0.267	< .001	0.227	0.306
age9edu	- Maths_ability_self_report_Lee2018	2117	0.091	< .001	0.048	0.133
age9edu	- Cognitive_performance_Lee2018	2117	0.270	< .001	0.230	0.309
age9edu	- Maths_highest_class_completed_Lee2018	2117	0.115	< .001	0.073	0.157
age9edu	- IQ_Savage2018_FRCT1	2117	0.269	< .001	0.229	0.308
age9edu	- slope	2117	-0.131	< .001	-0.172	-0.089

age9edu	- g_difference	2052	0.205	< .001	0.164	0.247
age12edu	- age16edu	2883	0.585	< .001	0.560	0.608
age12edu	- ases	2878	0.302	< .001	0.268	0.335
age12edu	- pcexgcseage1	2884	-0.170	< .001	-0.205	-0.135
age12edu	- difference_7	2988	0.185	< .001	0.151	0.220
age12edu	- EA4_no23andme_Okbay2022	2988	0.256	< .001	0.222	0.289
age12edu	- Maths_ability_self_report_Lee2018	2988	0.150	< .001	0.115	0.185
age12edu	- Cognitive_performance_Lee2018	2988	0.249	< .001	0.215	0.283
age12edu	- Maths_highest_class_completed_Lee2018	2988	0.172	< .001	0.137	0.207
age12edu	- IQ_Savage2018_FRCT1	2988	0.231	< .001	0.197	0.264
age12edu	- slope	2988	0.202	< .001	0.167	0.236
age12edu	- g_difference	1735	0.132	< .001	0.086	0.178
age16edu	- ases	3930	0.469	< .001	0.444	0.493
age16edu	- pcexgcseage1	4070	0.016	0.297	-0.014	0.047
age16edu	- difference_7	4070	0.131	< .001	0.101	0.162
age16edu	- EA4_no23andme_Okbay2022	4070	0.432	< .001	0.407	0.457
age16edu	- Maths_ability_self_report_Lee2018	4070	0.188	< .001	0.158	0.217
age16edu	- Cognitive_performance_Lee2018	4070	0.317	< .001	0.289	0.344
age16edu	- Maths_highest_class_completed_Lee2018	4070	0.229	< .001	0.200	0.258
age16edu	- IQ_Savage2018_FRCT1	4070	0.293	< .001	0.264	0.321
age16edu	- slope	4070	0.483	< .001	0.459	0.506
age16edu	- g_difference	2783	0.119	< .001	0.083	0.156
ases	- pcexgcseage1	3931	-0.022	0.160	-0.054	0.009
ases	- difference_7	4027	-0.093	< .001	-0.124	-0.063
ases	- EA4_no23andme_Okbay2022	4027	0.394	< .001	0.368	0.420
ases	- Maths_ability_self_report_Lee2018	4027	0.149	< .001	0.119	0.179
ases	- Cognitive_performance_Lee2018	4027	0.226	< .001	0.196	0.255
ases	- Maths_highest_class_completed_Lee2018	4027	0.199	< .001	0.169	0.229
ases	- IQ_Savage2018_FRCT1	4027	0.208	< .001	0.178	0.237

ases	- slope	4027	0.204	< .001	0.174	0.233
ases	- g_difference	2790	-0.006	0.750	-0.043	0.031
pcexgcseage1	- difference_7	4071	0.098	< .001	0.067	0.128
pcexgcseage1	- EA4_no23andme_Okbay2022	4071	-0.018	0.251	-0.049	0.013
pcexgcseage1	- Maths_ability_self_report_Lee2018	4071	-0.013	0.418	-0.043	0.018
pcexgcseage1	- Cognitive_performance_Lee2018	4071	-0.023	0.141	-0.054	0.008
pcexgcseage1	- Maths_highest_class_completed_Lee2018	4071	-0.019	0.233	-0.049	0.012
pcexgcseage1	- IQ_Savage2018_FRCT1	4071	-0.012	0.457	-0.042	0.019
pcexgcseage1	- slope	4071	-0.106	< .001	-0.137	-0.076
pcexgcseage1	- g_difference	2784	0.098	< .001	0.061	0.135
difference_7	- EA4_no23andme_Okbay2022	4175	-0.636	< .001	-0.654	-0.618
difference_7	- Maths_ability_self_report_Lee2018	4175	-0.188	< .001	-0.217	-0.159
difference_7	- Cognitive_performance_Lee2018	4175	-0.210	< .001	-0.239	-0.181
difference_7	- Maths_highest_class_completed_Lee2018	4175	-0.262	< .001	-0.290	-0.233
difference_7	- IQ_Savage2018_FRCT1	4175	-0.194	< .001	-0.223	-0.165
difference_7	- slope	4175	-0.456	< .001	-0.480	-0.432
difference_7	- g_difference	2884	0.377	< .001	0.345	0.408
EA4_no23andme_Okbay2022	- Maths_ability_self_report_Lee2018	4175	0.338	< .001	0.311	0.364
EA4_no23andme_Okbay2022	- Cognitive_performance_Lee2018	4175	0.495	< .001	0.472	0.518
EA4_no23andme_Okbay2022	- Maths_highest_class_completed_Lee2018	4175	0.443	< .001	0.418	0.467
EA4_no23andme_Okbay2022	- IQ_Savage2018_FRCT1	4175	0.467	< .001	0.443	0.490
EA4_no23andme_Okbay2022	- slope	4175	0.194	< .001	0.164	0.223
EA4_no23andme_Okbay2022	- g_difference	2884	0.034	0.072	-0.003	0.070
Maths_ability_self_report_Lee2018	- Cognitive_performance_Lee2018	4175	0.306	< .001	0.279	0.333
Maths_ability_self_report_Lee2018	- Maths_highest_class_completed_Lee2018	4175	0.862	< .001	0.854	0.869
Maths_ability_self_report_Lee2018	- IQ_Savage2018_FRCT1	4175	0.237	< .001	0.209	0.266
Maths_ability_self_report_Lee2018	- slope	4175	0.076	< .001	0.046	0.106
Maths_ability_self_report_Lee2018	- g_difference	2884	0.029	0.124	-0.008	0.065
Cognitive_performance_Lee2018	- Maths_highest_class_completed_Lee2018	4175	0.324	< .001	0.297	0.351

Cognitive_performance_Lee2018	- IQ_Savage2018_FRCT1	4175	0.799	< .001	0.788	0.810
Cognitive_performance_Lee2018	- slope	4175	0.068	< .001	0.038	0.098
Cognitive_performance_Lee2018	- g_difference	2884	0.056	0.003	0.020	0.092
Maths_highest_class_completed_Lee2018	- IQ_Savage2018_FRCT1	4175	0.265	< .001	0.237	0.293
Maths_highest_class_completed_Lee2018	- slope	4175	0.101	< .001	0.071	0.131
Maths_highest_class_completed_Lee2018	- g_difference	2884	0.029	0.122	-0.008	0.065
IQ_Savage2018_FRCT1	- slope	4175	0.050	0.001	0.020	0.081
IQ_Savage2018_FRCT1	- g_difference	2884	0.058	0.002	0.022	0.095
slope	- g_difference	2884	-0.394	< .001	-0.424	-0.363

Note: Correlation computed with pairwise exclusion. Legend: age7edu = Age 7 Achievement, age9edu = Age 9 Achievement, age12edu = Age 12 Achievement, age16edu = Age 16 Achievement, ases = SES, difference_7 = GPAΔ, EA4_no23andme_Okbay2022 = EA4 GPS, Maths_ability_self_report_Lee2018 = Math Ability GPS, Cognitive_performance_Lee2018 = Cognitive Performance GPS, Maths_highest_class_completed_Lee2018 = Math Attainment GPS, IQ_Savage2018_FRCT1 = Intelligence GPS, slope = Achievement Slope, g_difference = cogΔ.

Supplementary Figure 1. Scatterplot for the correlation of -0.44 between GPAΔ (calculated by residualizing achievement on GPS) and achievement slopes.

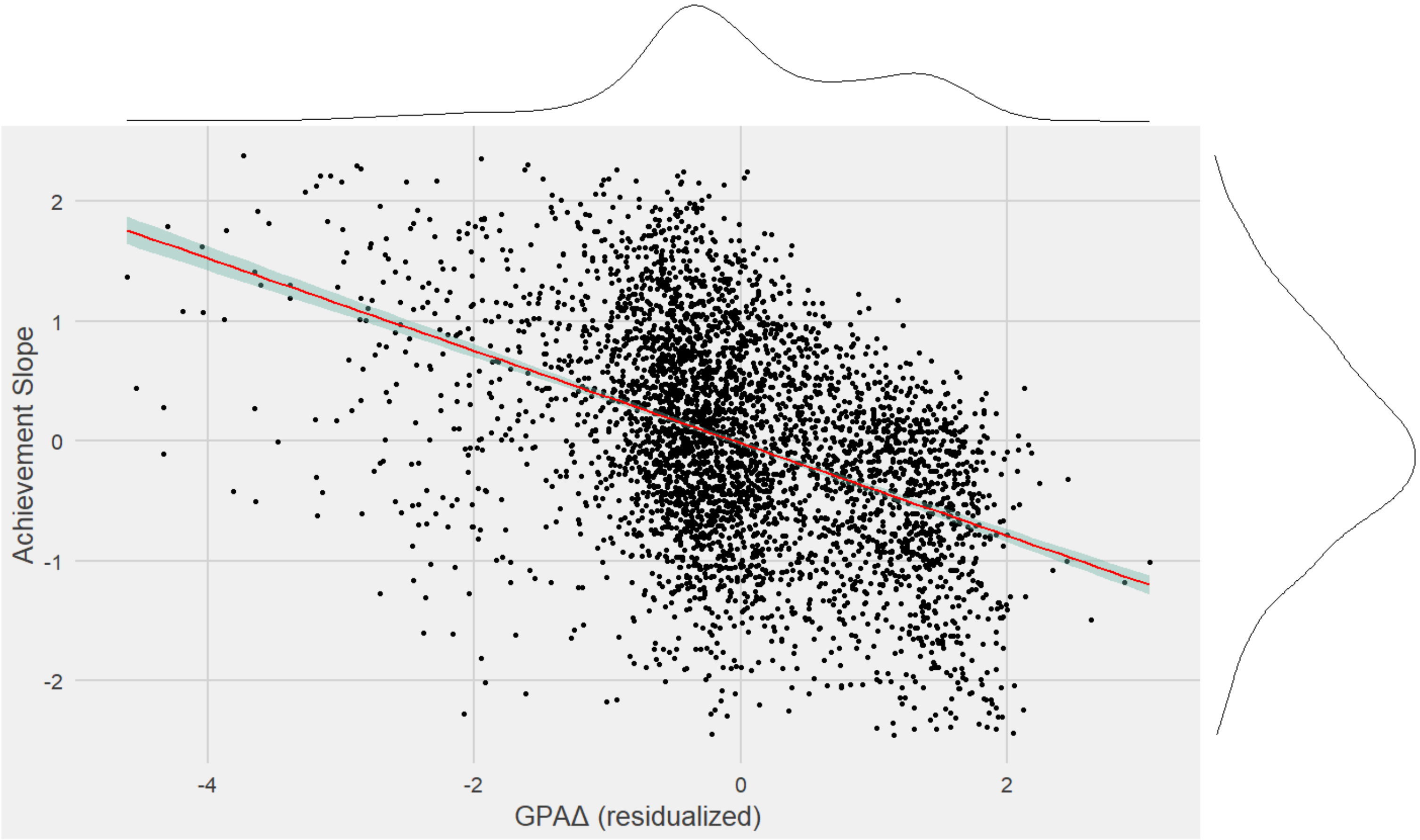

Supplementary Figure 2. Scatterplot for the correlation of 0.77 between GPAΔ (calculated by residualizing achievement on GPS) and GPAΔ (calculated by subtracting standardized GPS from achievement, as done in our study).

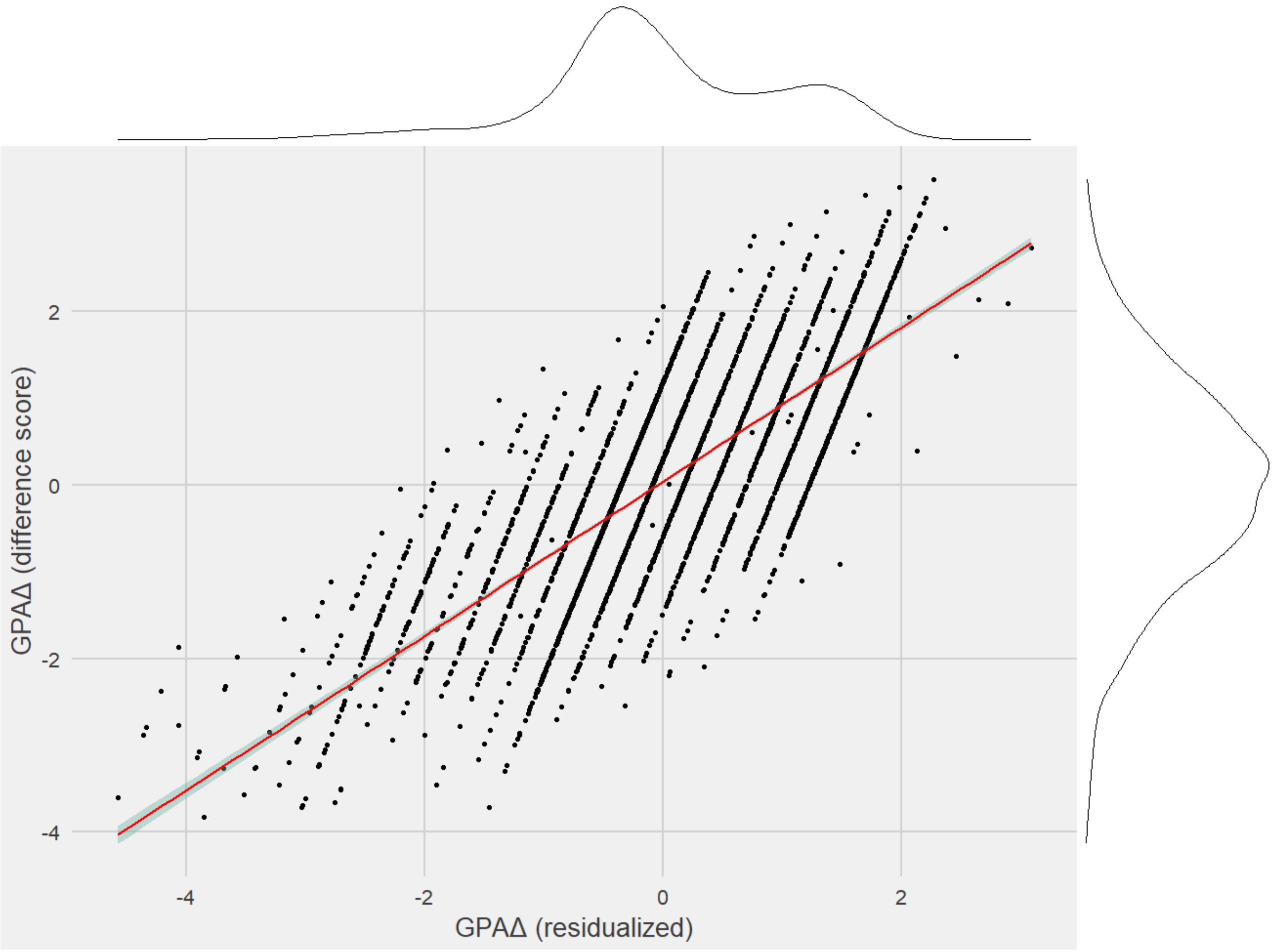

Supplement: Supplementary file 1 — Supplemental Material [file 41539_2024_251_MOESM1_ESM.pdf]
